# Natural Occurrence of *Escherichia coli*-Infecting Bacteriophages in Clinical Samples

**DOI:** 10.3389/fmicb.2019.02484

**Published:** 2019-10-31

**Authors:** Cátia Pacífico, Miriam Hilbert, Dmitrij Sofka, Nora Dinhopl, Ildiko-Julia Pap, Christoph Aspöck, João André Carriço, Friederike Hilbert

**Affiliations:** ^1^Department of Farm Animals and Veterinary Public Health, Institute of Food Safety, Food Technology and Veterinary Public Health, University of Veterinary Medicine, Vienna, Austria; ^2^Karl Landsteiner University of Health Sciences, Krems, Austria; ^3^Department of Pathobiology, University of Veterinary Medicine, Vienna, Austria; ^4^Institute of Hygiene and Microbiology, University Hospital St. Pölten, St. Pölten, Austria; ^5^Faculty of Medicine, Instituto de Medicina Molecular, University of Lisbon, Lisbon, Portugal

**Keywords:** bacteriophages, urine, tracheal aspirates, blood, *E. coli*, *Peduovirus*, *Tunavirinae*, *Tequintavirus*

## Abstract

The interaction between bacteriophages, bacteria and the human host as a tripartite system has recently captured attention. The taxonomic diversity of bacteriophages, as a natural parasite of bacteria, still remains obscure in human body biomes, representing a so-called “viral dark matter.” Here, we isolated and characterized coliphages from blood, urine and tracheal aspirates samples collected at a tertiary care hospital in Austria. Phages were more often isolated from blood, followed by urine and tracheal aspirates. Phylogenetic analysis and genome comparisons allowed the identification of phages belonging to the *Tunavirinae* subfamily, and to the *Peduovirus* and *Tequintavirus* genera. *Tunavirinae* phages cluster together and are found in samples from 14 patients, suggesting their prevalence across a variety of human samples. When compared with other phage genomes, the highest similarity level was at 87.69% average nucleotide identity (ANI), which suggests that these are in fact a newly isolated phage species. *Tequintavirus* phages share a 95.90% with phage 3_29, challenging the ANI threshold currently accepted to differentiate phage species. The isolated phages appear to be virulent, with the exception of the *Peduovirus* members, which are integrative and seem to reside as prophages in bacterial genomes.

## Introduction

*E. coli*, a bacterium that inhabits the gastrointestinal tract of humans and warm-blooded animals, is the most prevalent commensal bacteria. It represents the most frequent cause of infections in healthcare settings and in the community, and it is the reason for most bloodstream infections in Europe (Weinbauer, 2004). Additionally, it is involved in the development of bacterial diseases such as enteritis, neonatal meningitis, urinary tract infections, bloodstream infections and intra-abdominal infections (Allocati et al., 2013; European Centre for Disease Prevention and Control, 2016).

It had been previously demonstrated that phages have an impact on bacterial pathogenesis by carrying virulence factors, antimicrobial resistance genes, and host adaptation factors (Brussow et al., 2004). As important vehicles of horizontal gene transfer, phages contribute significantly to the strain-to-strain differences observed within the same bacterial species (Desiere et al., 2001). They tend to infect specific isolates or groups of isolates of closely related species, particularly in the *Enterobacteriaceae* family, but some viruses can infect members of several genera (King et al., 2012). A predominant narrow host-range keeps the bacterial populations under control, retaining the balance of competitively dominant species or populations (Weinbauer, 2004).

Phages are typically found and transmitted in terrestrial and aqueous environments, even though they can be retrieved in any environment where their bacterial hosts are present (Weinbauer, 2004; McNair et al., 2012). Coliphages – bacteriophages that target specifically *Escherichia coli* strains – have been used as indicator for fecal coliforms and enteric viruses for water pollution (International Organization for Standardization, 2000). In this context, coliphages have been isolated in clinical settings from hospital effluents (Bibi et al., 2016; Latz et al., 2017; Peng and Yuan, 2018) and human body biomes such as the gastrointestinal tract, oral cavity, saliva, sputum, and urine (Górski and Weber-Dabrowska, 2005; Hyman and Abedon, 2012). The human body, as an ecosystem, consists of a significant amount of viral dark matter (Kowarsky et al., 2017; Thannesberger et al., 2017). This refers to the amount of unidentified phage sequences, estimated around 85% in the human gut while known prophages and temperate phages comprise the remaining part. In comparison to the high diversity in free-living bacterial communities, the number of phages per bacterial species in the human gut is reported to be quite reduced (Reyes et al., 2010). These phages are thought to be associated with the bacteria in the background microbial flora (Kowarsky et al., 2017). Metagenomics studies confirmed that phages appear to dominate the human body virome regardless of eukaryotic viruses (Reyes et al., 2010; Hyman and Abedon, 2012) and revealed that the predominant viral group of human fecal samples is siphoviruses (Breitbart et al., 2003), the most prevalent group in natural habitats (Weinbauer, 2004).

The occurrence of phages in the human body raises questions regarding their importance in physiology and pathology and in their interaction with the pre-existing microbial communities. In fact, prophages and phage-like sequences have been shown to contribute to 20% of the bacterial genomes (Wang et al., 2010). Given that a lot of uncultured viruses and metagenomic sequences lack morphological characterization, which is a key criteria for the classification by the International Committee on the Taxonomy of Viruses (ICTV), this study aimed to characterize the viral diversity underneath the viral dark matter that circulates in different human body habitats of clinical relevance.

## Materials and Methods

### Sample Collection

The specimens included in this survey were anonymized leftovers from samples collected according to institutional standards for routine microbiological testing at an Austrian tertiary care hospital, and submitted to the local Institute of Hygiene and Microbiology. Between October 2017 and August 2018, a total of 111 human fluid samples (abdominal, lung, pleural, shoulder and knee effusions, transport fluid from nasal swabs and intravenous catheters, blood, tracheal aspirates and urine) were thus collected. After diagnostic routine, leftovers with a volume between 3 and 5 ml were placed in a sterile tube to be processed for further analysis preferably at the day of collection. Whenever this was not possible samples were stored at 4°C for a maximum of 1 day to avoid a decrease in phage viability. Priority was given to samples susceptive to the presence of *E. coli* or staphylococci. Trachea aspirates are collected for routine investigation of the respiratory fluids and urine samples were collected for testing of *Legionella* and pneumococci-antigen. The collection was done in four sampling rounds (I–IV) (Table 1). The samples were then further processed in a BSL-2 laboratory in our facilities. No personal data or any other information than the type of material, the date of collection and the result of routine microbiology analysis was associated with each specimen, inhibiting any correlation of these fully anonymized samples to the respective patients. Thus, according to national regulations and the institutional rules for Good Scientific Practice, the requirement for submission to an ethical committee and for obtaining patients’ informed consent was waived.

**TABLE 1:** Sampling scheme containing the type of biological fluid samples (urine, blood, respiratory tract fluids, and others) obtained during each of the four sampling rounds (I–IV), in a total of 111 samples analyzed.

**Sampling round**	**Collection date**	**Type of sample (*n*)**				
		
		**Urine**	**Blood**	**Respiratory tract fluids**	**Others (e.g., abdominal, lung, pleural, shoulder and knee fluid, nasal swabs, IV catheter)**	**Total**
I^a^	14/11/17	12	2	4	5	23
II	28/01/18	12	6	9	0	27
III	26/03/18	11	6	7	8	32
IV	22/08/18	9	7	13	0	29
Total		44	21	33	13	111

**^*a*^The samples collected during this sampling round were not tested for bacteria.**

### Isolation of *Escherichia coli* Colonies

An aliquot of 50 μl of each sample was spread onto Coli-ID agar plates (bioMérieux, Marcy l’Etoile, France) and incubated at 37°C. Colony-forming units (CFUs) were counted at 24 and 48 h. Five colonies of each sample were further sub-cultured on Modified Scholtens’ Agar (MSA) plates and incubated 24 h at 37°C. The isolates were stocked in 20% (wt/vol) glycerol (Sigma-Aldrich, St. Louis, MO, United States) at −80°C.

### Bacterial Host, Culture Conditions, and Bacteriophage Isolation

Samples were filtered using a 0.22 μm filter to remove bacterial load and processed using the soft-agar overlay method according to standard ISO 10706-2:2000, as previously described (Shousha et al., 2015; Hilbert et al., 2017) but with slight modifications. Briefly, three milliliters of semi-solid Modified Scholtens’ Agar (ssMSA) were preheated to 45°C and supplemented with 10 mM CaCl_2_. An overnight culture of the indicator strain was used to inoculate a new culture and was grown to an optical density of 0.4 at 600 nm. One milliliter of host culture and 1 ml of sample were added to the preheated semi-solid MSB and the mixture was vortexed and overlaid on MSA at room temperature and after solidifying, incubated overnight at 37°C. *E. coli* DSM 12242 was used as indicator host strain for bacteriophage detection. The filtered sample was either plated directly with the host bacteria, or spotted in an overlay, or mixed with the host bacteria in a liquid culture with further plating of the resulting supernatant. Lysates of plaques (five per sample whenever possible) were prepared according to Groisman (Groisman, 1991) by individually picking plaques from the soft agar and suspending them in 1 ml MSB media. The solution was then incubated 30 min at 37°C and 250 rpm. After centrifugation at 10,000 *g* during 8 min, the lysates were filtered through a 0.22 μm-pore-size filter and stored at 4°C. Blood samples were pre-processed prior to filtration. These samples were centrifuged at 10,000 rpm during 5 min and the upper layer was set aside, followed by a second centrifugation at the same conditions. The resulting supernatant was then filtered and used as described above.

### Statistical Analysis

Statistical analysis was performed using Fisher’s exact test to investigate the association between the absence/presence of *E. coli* in the body fluids analyzed and the absence/presence of lytic coliphages. The significance level was set to a level of *p* < 0.05.

### Preparation of Concentrated Lysates

Bacteriophage suspensions were propagated by re-infection the indicator host in triplicate using the purified lysate and the soft agar overlay method. After overnight incubation at 37°C, the soft agar was shredded and 3 ml of media were added. The overlay was collected and centrifuged at 8,000 *g* during 5 min. The clear supernatant was filtered through a 0.22 μm-pore-size filter and kept at 4°C. Ten-fold serial dilutions (10^–1^ to 10^–6^) were prepared by plating 10 μl of the diluted lysate with the indicator bacteria. After overnight incubation at 37°C, the plaques were counted and the titer was expressed as PFU/ml.

### Transmission Electron Microscopy (TEM)

A droplet of the purified phage suspension (10^7^–10^8^ PFU/ml) was deposited on a copper grid (Science Services, Munich, Germany) with carbon-coated Formvar film for 10 min at room temperature and stained with 4% aqueous phosphotungstic acid (Merck, Darmstadt, Germany) at pH 7. The sample was air-dried overnight and analyzed with a Zeiss TEM 900 electron microscope (Carl Zeiss, Oberkochen, Germany) operated at 50 kV. The phage particles were visualized using the Image SP software and a CCD camera (TRS, Tröndle Restlichtverstärkersysteme, Moorenweis, Germany).

### Host Range Analysis and Efficiency of Plating

The laboratory host bacteria *E. coli* DSM 12242, ATCC 11303, JM109, W3110, MC1061, and DH5α and other members of the *Enterobacteriaceae* family were used to assess the lytic spectra of the phages. Clinical isolates from our own bacterial collection and from the clinical samples analyzed in this study were included in the screening (Table 2). Ten microliters of concentrated phage lysates (10^8^ PFU/ml) were spotted onto MSA plates previously overlaid with 100 μl of stationary phase cell suspensions mixed with 3 ml of Molten-soft agar supplemented with 10 mM CaCl_2_. The plates were incubated overnight at 37°C. Whenever lysis was observed, the efficiency of plating (EOP) was calculated. EOP was defined as the ratio between PFU/ml on the sensitive bacteria and the PFU/ml on the indicator strain.

**TABLE 2:** Bacterial strains used to characterize the lysis spectra of the phages and respective EOP.

**Bacteria**	**Phage group**			**References**
		
	***Peduovirus* vB_EcoM-12474V**	***Tequintavirus* vB_EcoS-26175V**	***Tunavirinae* vB_EcoS-2004IV**	
*Escherichia coli* DSM 12242	1.000 ± 0.000	1.000 ± 0.000	1.000 ± 0.000	DSMZ
*Escherichia coli* ATCC 11303	–	0.228 ± 0.048	–	ATCC
*Escherichia coli* W3110 (ATCC 27325)	–	0.840 ± 0.260	0.812 ± 0.080	ATCC
*Escherichia coli* JM109 (DSM 3423)	–	0.011 ± 0.004	0.145 ± 0.030	DSMZ
*Escherichia coli* DH5α (DSM 6897)	–	0.195 ± 0.055	–	DSMZ
*Escherichia coli* MC1061 (ATCC 53338)	0.283 ± 0.150	0.541 ± 0.287	0.509 ± 0.034	ATCC
*Klebsiella pneumoniae sub. pneumoniae* ATCC 13883	–	–	–	ATCC
*Yersinia enterocolitica sub. palearctica* DSM 11502	–	–	–	DSMZ
*Salmonella enterica sub. enterica* ATCC 14028	–	–	–	ATCC
*Salmonella typhimurium* DT104 isolate H3380	–	–	–	Clinical (Briggs and Fratamico, 1999)
*Escherichia coli* isolated from samples (n = 18)	–	–	–	This study
*Escherichia coli* isolates (*n* = 20)	–	–	–	Clinical
*Escherichia coli* isolate *P3*	–	–	–	Clinical
*Escherichia coli* isolate *P4*	–	–	–	Clinical
*Escherichia coli* isolate *P5*	–	–	–	Clinical
*Klebsiella pneumoniae* isolate P6	–	–	–	Clinical
*Klebsiella pneumoniae* isolate P7	–	–	–	Clinical
*Klebsiella pneumoniae* isolate P8	–	–	–	Clinical
*Klebsiella oxytoca* isolate P10	–	–	–	Clinical

**These consisted in E. coli laboratory strains used in phage enumeration and isolation, additional Enterobacteriaceae species, E. coli isolated from the biological fluid samples and other bacterial isolates collected from patients and outpatients in this clinical setting.**

### Tolerance to Disinfectants

Phage stability was determined in the presence of 0.25% commonly used hospital disinfectants TPH Protect and Hexaquart^®^ plus. Phage preparations of 10^7^ PFU/ml were incubated at RT and phage titer was determined at 0, 15, 30, and 60 min by serial dilutions tested on the indicator strain by means of the soft agar overlay method, as before (Salem and Skurnik, 2018). The phage particles were counted after overnight incubation at 37°C. Assays were performed in duplicate. All the tested conditions allowed the survival and normal growth of the host strain, not hindering the phage infection.

### Bacteriophage DNA Extraction

DNA was isolated using a phenol-chloroform extraction method with some modifications. Concentrated phage suspensions were pretreated with DNase I (Quantabio, Beverly, MA, United States) and RNase A (Sigma-Aldrich, St. Louis, MO, United States) in 10 μg/ml final concentration and the mixture was inverted several times and incubated at 37°C during 30 min. Furthermore, 20 mg/ml proteinase K (40 μg) and a final concentration of 0.5% sodium dodecyl sulfate (SDS) were added and incubated at 65°C for 1 h. The suspension was cooled to RT and phenol:chloroform extraction was performed, followed by an ethanol precipitation. The precipitated DNA was dissolved in 200 μl ddH_2_O. The DNA concentration was estimated based on the band intensity comparison to the 1 kb marker (peqGOLD 1 kb DNA-Ladder, PEQLAB Biotechnologie GmbH, Austria). Whenever the DNA concentration proved to be insufficient, ethanol precipitation was repeated using ammonium acetate at a final concentration of 2.0–2.5 M. The DNA was restricted with enzyme *Pvu*II (Roche Diagnostics GmbH, Vienna, Austria) using manufacturer’s protocol and separated on a 1% agarose gel as an attempt to distinguish different restriction profiles.

### Whole Genome Sequencing (WGS)

All library preparation, DNA sequencing and sequence trimming were performed at MicrobesNG (Birmingham, United Kingdom). Genomic libraries were prepared using the Nextera XT Library Prep Kit (Illumina, San Diego, CA, United States) according to manufacturer’s instructions with slight modifications. Libraries were sequenced on the Illumina HiSeq using 250 bp paired-ends approach. The trimmed reads were analyzed using the INNUca pipeline (Machado et al., 2016). Briefly, reads quality were improved using Trimmomatic v0.38 (Bolger et al., 2014) and assembly was performed using Pear v0.9.10 and SPAdes version 3.13 (Bankevich et al., 2012). Resulting assembly was corrected by Pilon v1.18 and contigs were annotated using Prokka 1.13.3 (Seemann, 2014). Hypothetical proteins were manually annotated by using blastp against viral databases (Supplementary Data S2–S4). Contigs having a coverage lower than 1/3 of the assembly and smaller than 200 bp were considered as putative artifacts/contaminations and therefore removed from the downstream analysis. Genomes yielding a coverage <15x were excluded from the analysis.

### Bioinformatics and Comparative Genome Analysis

The taxonomic distribution was assessed using Kraken v1 (Wood and Salzberg, 2014). Phylogenetic analysis of the 43 bacteriophages was re-constructed using VICTOR^1^ (Meier-Kolthoff et al., 2013) with recommended settings for prokaryotic viruses (Meier-Kolthoff and Göker, 2017). The presence of antimicrobial resistance determinants such as antibiotic resistance and virulence genes was investigated using the command-line tool ABRicate v0.8.10^2^. Genome maps were constructed using the web-based tool GenomeVx (Conant and Wolfe, 2008). Average nucleotide identity (ANI) was calculated using OrthoANI^3^ (Ha et al., 2017). The amino acid sequences of the integrase (*Peduovirus*), the large subunit terminase protein (*Tunavirinae*) and the DNA polymerase (*Tequintavirus*) were used for phylogenetic analysis. Sequence alignment was determined using Clustal Omega (Sievers et al., 2011; Chojnacki et al., 2017) and trees were reconstructed applying a bootstrapped (500 replications) Maximum Likelihood (ML) analysis. Tree inference used a Nearest-Neighbor-Interchange (NNI) as a heuristic method and a Jones-Thaylor-Thornton (JTT) as substitution model. Bacteriophages were compared with complete phage genomes deposited in the NCBI Viruses database for *Peduovirus*, *Tequintavirus*, and subfamily *Tunavirinae* (April 2019). Accession numbers and ANI% of the closest homologs according to MASH^4^ are given in the Supplementary Data S5. Evolutionary analysis were carried out using MEGA X v10.0.5 (Kumar et al., 2018) and trees were visualized and edited with FigTree v1.4.4^5^.

### Sequence Accession Numbers

Raw sequence data were deposited in the ENA under project accession number PRJEB32459. Assembled genomes and annotations were directly submitted to NCBI (PRJNA541793). Accession numbers are given in the Supplementary Data S1.

## Results

### Phage, Bacteria, and Prevalence in Clinical Samples

A total of 111 samples were screened for the presence of lytic bacteriophages infecting *E. coli* DSM 12242 (Table 1). Coliphages were detected in 16 samples, representing a prevalence of 14.4% in the body fluids analyzed. Phages were more commonly found in blood (23.8%), followed by urine (20.5%) and tracheal aspirates (6.1%). No coliphages were found in other samples analyzed (abdominal, lung, pleural, shoulder and knee fluid, nasal swabs, IV catheters). From 88 samples, 16 were positive for the presence of *E. coli*, with bacterial counts ranging between 1,65 × 10^7^ and 3,45 × 10^12^ CFU/ml. From these 16 samples, in 5 of them lytic phages were concomitantly isolated. In fact, a statistically significant association between the absence/presence of *E. coli* and the presence/absence of coliphages in the sample was determined (*p* = 0.016).

### Bacteriophage Diversity

**FIGURE 1:**
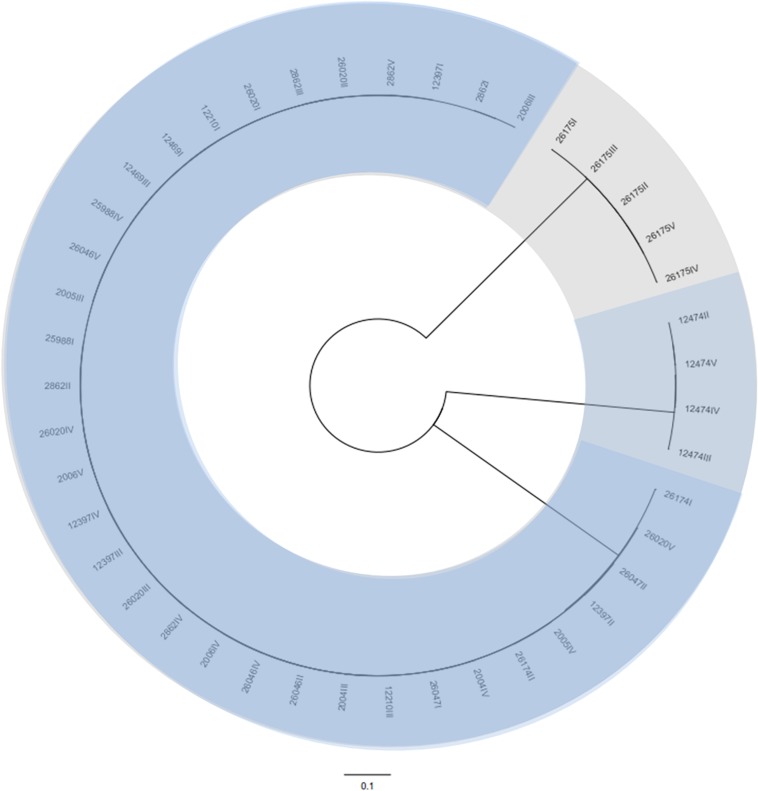
Phylogenetic tree of the 43 bacteriophages isolated during this study using VICTOR (Supplementary Data S1). The scale represents homology % and yields an average support of 33%. The number of clusters determined were three, representing the genetic groups here described – *Peduovirus* (mild blue), *Tequintavirus* (gray) and *Tunavirinae* (blue).

A total of 43 coliphages isolated from 16 human body fluid samples were purified, sequenced and analyzed (Table 3). Three distinct genetic groups of linear double-stranded DNA viruses were identified using both a Genome-BLAST Distance Phylogeny (GBDP) method and a MASH all-versus-all approach, where phage genomes were compared against each other and grouped based on sequence similarity (Figure 1). This genomic diversity was also in accordance with the output of Kraken, which evidenced the presence of three phage groups belonging to the “P2likevirus,” “T5likevirus,” and “Tunalikevirus.” One representative phage of each genetic group was further selected for more in-depth analysis.

**TABLE 3:** Description of the isolation source, morphology, and sequence characteristics of the 43 bacteriophages sequenced in the course of this study.

**Phages analyzed**	**Sampling round**	**Isolation source**	**Clinical history and microbiological findings**	**Morphology**	**Genome characteristics**			
					
					**Size (Kb)**	**GC content (%)**	**CDS (n)**	**tRNAs (n)**
vB_EcoS-2862I, vB_EcoS-2862II, vB_EcoS-2862III, vB_EcoS-2862IV, vB_EcoS-2862V	II	Tracheal aspirate	*Klebsiella variicola*^∗^	*Siphoviridae*	44.2	44.4–44.5	65–66	1
vB_EcoS-26020I, vB_EcoS-26020II, vB_EcoS-26020III, vB_EcoS-26020IV, vB_EcoS-26020V	I	Urine	^∗∗^	*Siphoviridae*	44.2	44.4–44.5	65–67	1
vB_EcoS-12469II, vB_EcoS-12469III	III	Blood	Urinary tract infection (*E. coli, Bacteroides fragilis*)	*Siphoviridae*	44.2	44.4	66	1
vB_EcoS-12210I, vB_EcoS-12210III	III	Blood	Status febrilis (*S. aureus*)	*Siphoviridae*	44.2	44.4	64–65	1
vB_EcoS-2005III, vB_EcoS-2005IV	II	Urine	^∗∗^	*Siphoviridae*	44.2	44.4	66	1
vB_EcoS-26046II, vB_EcoS-26046III, vB_EcoS-26046IV, vB_EcoS-26046V	I	Urine	^∗∗^	*Siphoviridae*	44.2	44.4	66	1
vB_EcoS-2006III, vB_EcoS-2006IV, vB_EcoS-2006V	II	Urine	^∗∗^	*Siphoviridae*	44.2	44.4	66	1
vB_EcoM-12474II, vB_EcoM-12474III, vB_EcoM-12474IV, vB_EcoM-12474V	III	Blood	Urinary tract infection (*E. coli*)	*Myoviridae*	33.7–33.8	50.9	44	0
vB_EcoS-2004III, vB_EcoS-2004IV	II	Urine	^∗∗^	*Siphoviridae*	44.2	44.4	66	1
vB_EcoS-25988I, vB_EcoS-25988IV	I	Urine	^∗∗^	*Siphoviridae*	44.2	44.4	65–66	1
vB_EcoS-26174I, vB_EcoS-26174II	I	Urine	^∗∗^	*Siphoviridae*	44.2	44.4	65–66	1
vB_EcoS-26175I, vB_EcoS-26175II, vB_EcoS-26175III, vB_EcoS-26175IV, vB_EcoS-26175V	I	Urine	^∗∗^	*Siphoviridae*	114.6	39.9–40.0	158	31
vB_EcoS-26047I, vB_EcoS-26047II	I	Urine	^∗∗^	*Siphoviridae*	44.2	44.4	65–66	1
vB_EcoS-12397I, vB_EcoS-12397II, vB_EcoS-12397III, vB_EcoS-12397IV	III	Tracheal aspirate	Oropharyngeal flora and *Candida tropicalis ^∗^*	*Siphoviridae*	44.2	44.4	65–66	1

*^∗^routine investigation of respiratory tract fluids, clinical history most likely not because of infectious disease; ^∗∗^all urine samples were not tested for bacterial culture but for Legionella or pneumococci-antigen only.*

### Basic Genome Characteristics and Phylogenetic Analysis

**FIGURE 2:**
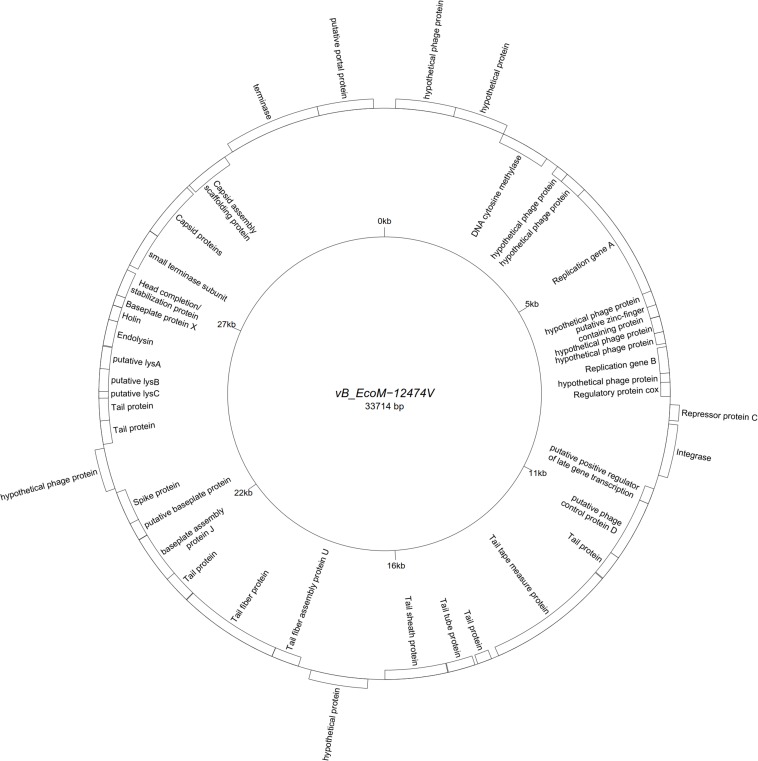
Genome map of the representative *Peduovirus* phage vB_EcoM-12474V (MK907239) isolated during this study.

All assemblies generated one single contig, with the exception of vB_EcoS-26174I (Table 3). The genome of the four “P2likevirus” phages (vB_EcoM-12474II, III, IV, and V) is between 33,688 and 33,807 bp long, contains 44 protein-encoding CDS and no tRNA genes. The GC content is 50.9%. All four “P2likevirus” bacteriophages were isolated from the same blood sample and shared an ANI of 100%. From the 44 proteins predicted, 28 were initially deemed as hypothetical. After carefully inspecting each protein and using blastp searches, only two proteins were unique in our phages and had no homology in the NCBI viral database. The remaining proteins were annotated or designated as “hypothetical phage proteins” whenever homology in other phage sequences was found (Figure 2). Phage attachment sites, attL (CCCGCCCGCTTCATGGGTCGGTTTTAATG) and attR (CCCGCCCGCTTCATGGGTCGGTTTTAATG) were also detected in the sequence.

**FIGURE 3:**
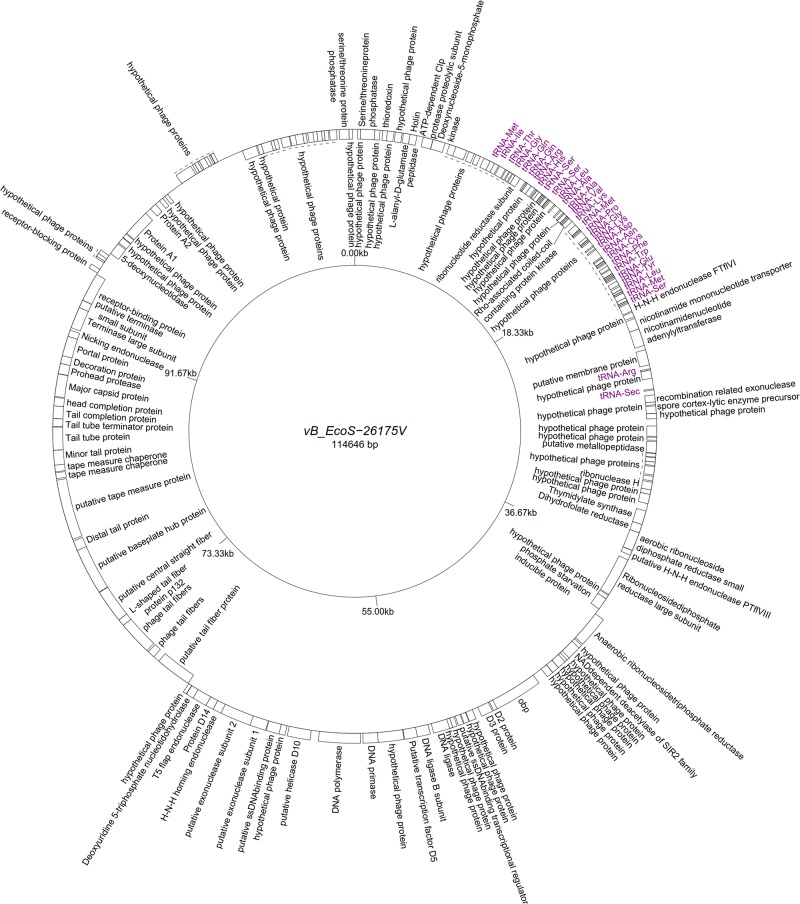
Genome map of the representative *Tequintavirus* phage vB_EcoS-26175V (MK907271) isolated during this study.

**FIGURE 4:**
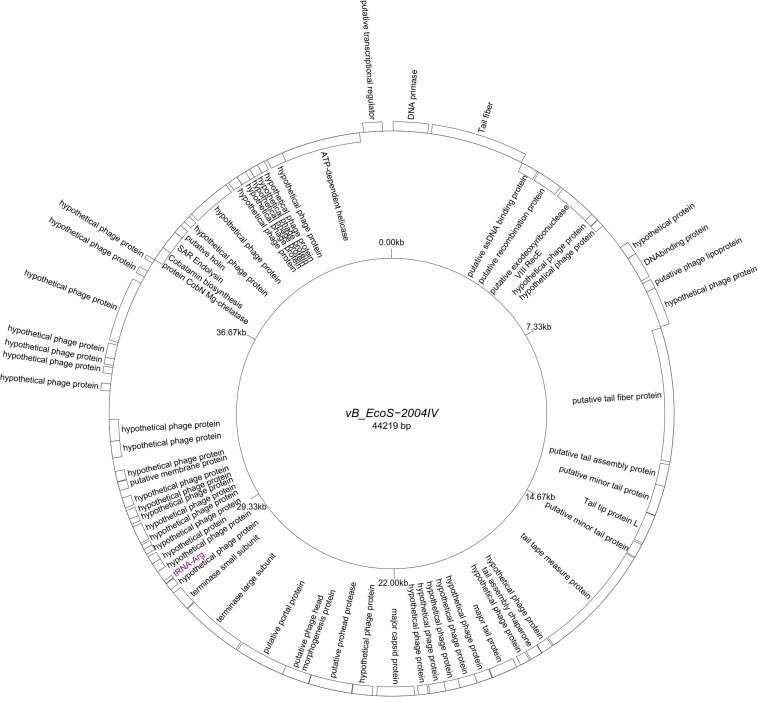
Genome map of the representative *Tunavirinae* phage vB_EcoS-2004IV (MK907241) isolated during this study.

Five “T5likevirus” phages were isolated during this study from a urine sample, sharing a 99.93–100% nucleotide sequence homology among themselves. Phages vB_EcoS-26175 I, II, III, IV, and V have a genome length of 114,646–114,649 bp and between 39.9 and 40.0% GC content. These phages have 158 putative CDS and 31 tRNA genes. Eighty-seven phage-related hypothetical proteins were found and two have not been described in the databases used (Figure 3). The remaining 34 phages were classified as “Tunalikevirus” and had a genome of 44,219 bp, 64–68 CDS and 1 tRNA gene. From these, 38 were phage-related hypothetical proteins with homologs in other virus genomes in the database and two are unique to these sequences (Figure 4). The GC content was between 44.4 and 44.5%.

No bacterial antimicrobial resistance gene, toxin or virulence determinant were found in the phage genomes.

### *Peduovirus* (“P2likevirus”)

**FIGURE 5:**
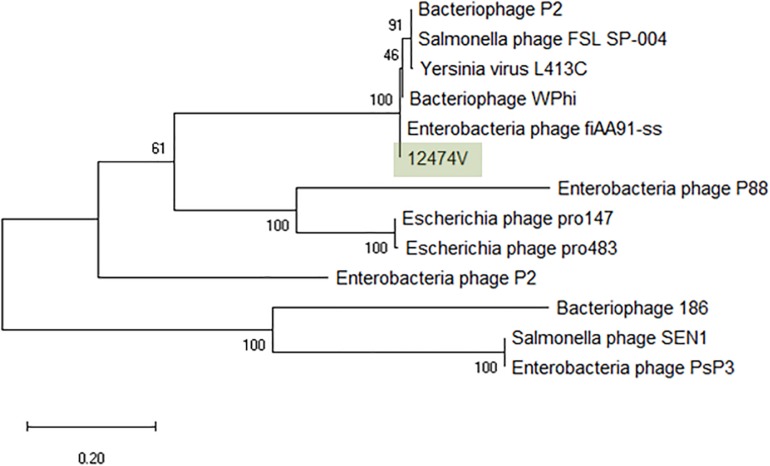
Phylogenetic tree of *Peduovirus* phage based on the Clustal Omega alignment of the integrase protein sequence of the representative phage vB_EcoM-12474V (MK907239). The percentage of trees in which the associated taxa clustered together is shown next to the branches. The list of genomes included and GenBank accession numbers are described in Supplementary Data S5.

The greatest nucleotide similarity level identified with vB_EcoM-12474V were phages fiAA91-ss (NC_022750) and pro483 (NC_028943), both members of the *Peduovirus* genus. Similarity to bacteriophage P2 (NC_001895), representative of this genus, was 96.59%. The phylogeny of these phages was further investigated by comparison of the amino acid sequence of the integrase (367 amino acids) (Figure 5). The *Peduovirus* isolated in this study shares a 97.30% whole genome ANI with fiAA91-ss (NC_022750). These phages are closely associated with bacteriophage WPhi (NC_005056), L-413C (NC_004745), and P2 (NC_001895), all officially classified as *Peduovirus* by the ICTV. Whole-genome based phylogeny of all *Peduovirus* clearly corroborates this data (Supplementary Figure S1) and adds pro147 (NC_028896) and pro483 (NC_028943), also classified *Peduovirus*, as closest homologs. In fact, the ANI% of vB_EcoM-12474V is ≥95% with these seven phages, suggesting they are representatives of one of these species. An alignment of the P*eduovirus* genomes shows non-collinearization of phage genomes and almost no order conservation of the genetic blocks. Nonetheless the synteny seems to be maintained (Supplementary Figure S2).

### *Tequintavirus* (“T5likevirus”)

**FIGURE 6:**
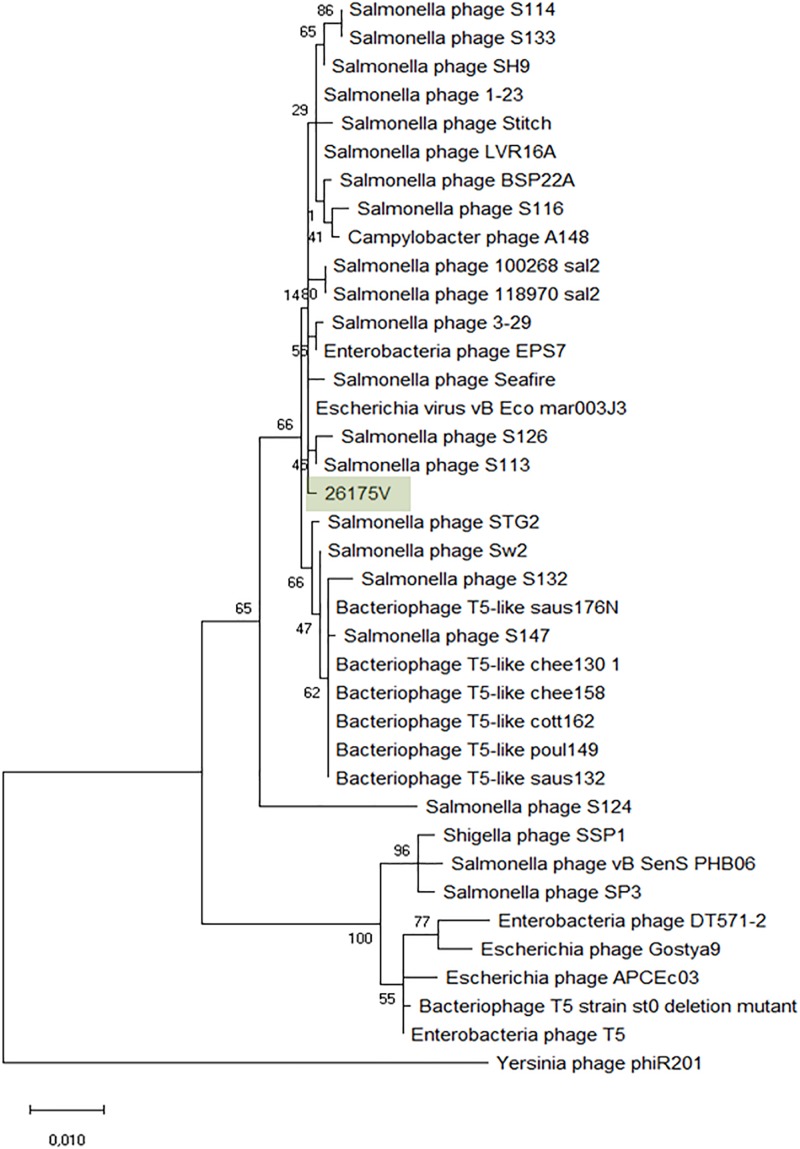
Phylogenetic tree of *Tequintavirus* phage based on the Clustal Omega alignment of the DNA polymerase I protein sequence of the representative phage vB_EcoS-26175V (MK907271). The percentage of trees in which the associated taxa clustered together is shown next to the branches. The list of genomes included and GenBank accession numbers are described in Supplementary Data S5.

The *Tequintavirus* genus belongs to the *Siphoviridae* family. All the phages currently listed as part of this genus were extracted from Genbank. Greatest similarity to vB_EcoS-26175V was found to phage vB_Eco_mar003J3 (LR027389), S126 (MH370376), 3-29 (MK393882), A148 (MG065642), and 118970_sal2 (NC_031933), and Stitch (NC_027297) based on MASH identity. MASH identified at least 34 phages as closest homologs and from these, 27 having at least 93.11% ANI to our phages (Supplementary Figure S3). Nucleotide similarity to phage T5 (NC_005859) was only 82.79% and therefore it is not the same species (%ANI ≥ 95). ANI with 118970_sal2 and Stitch was 95.08 and 95.45% respectively, but the closest nucleotide identity was 95.90% with phage 3–29. The phylogeny of these phages was further investigated by comparison of the amino acid sequence of the DNA polymerase (1216 amino acids), as previously (Sváb et al., 2018; Michniewski et al., 2019; Figure 6). Phages S126 and S113 (MH370366) appear closer to 26175V. The phages were deposited under the name *Escherichia* phage vB_EcoS-26175I, vB_EcoS-26175II, vB_EcoS-26175III, vB_EcoS-26175IV, and vB_EcoS-26175V and their gene content was compared with the closest homologs (Supplementary Figure S4).

### Tunavirinae

**FIGURE 7:**
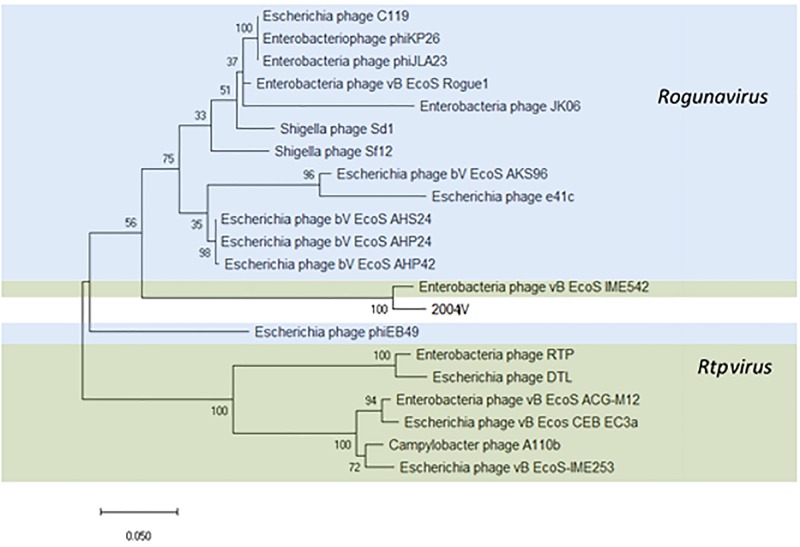
Phylogenetic tree of *Tunavirinae* phage based on the Clustal Omega alignment of the terminase large subunit protein sequence of the representative phage vB_EcoS-2004IV (MK907241). Phages currently classified as Rogunavirus and as Rtpvirus are highlighted in blue and green, respectively. The percentage of trees in which the associated taxa clustered together is shown next to the branches. The list of genomes included and GenBank accession numbers are described in Supplementary Data S5.

Thirty-four identical phages from 14 samples were identified in this study as putative “Tunalikevirus” according to Kraken, but whole-genome sequence analysis puts them closer to the members of the *Rtpvirus* genus (Supplementary Figure S5). To classify the phages identified in this study, the amino acid sequence of the large subunit terminase protein (534 amino acids) was chosen (Kropinski et al., 2015; Michniewski et al., 2019) and compared with the same sequence of 20 *Tunavirinae* members of both *Rtpvirus* and *Rogunavirus* genera identified as closest homologs using MASH, all having an ANI% ≥ 66.94 (Figure 7). According to the amino acid sequence of the terminase large subunit protein, both vB_EcoS_IME542 (MK372342) and the new *Tunavirinae* phages isolated in this study form a clade closer to the *Rogunavirus* genus. Phage vB_EcoS_IME542 is not classified by the ICTV but rather shows up in the NCBI database as an *Rtpvirus* member. In regards to ANI, the most similar phage genomes at the nucleotide level are the *Rtpvirus* phages A110b (MG065688), followed by the vB_Ecos_CEB_EC3a (KY398841), vB_EcoS-IME253 (KX130960), vB_EcoS_ACG-M12 (NC_019404), DTL (MG050172), RTP (NC_007603), and vB_EcoS_IME542. When looking at the amino acid sequence of the large subunit terminase, both vB_EcoS_IME542 and the newly isolated phage vB_EcoS-2004IV are closer to *Rogunavirus*. Both share a 96.37% amino acid identity at the level of the large subunit terminase. Whole genome analysis and ANI% places them both closer to the *Rtpvirus*. The most similar phage is A110b, a non-classified phage also closer to the *Rtpvirus* genus according to whole-genome phylogeny reconstruction, with an 87.69% ANI in common with the new phages, the highest value obtained when scanning the database (Supplementary Figure S6).

### Bacteriophage Characterization

**FIGURE 8:**
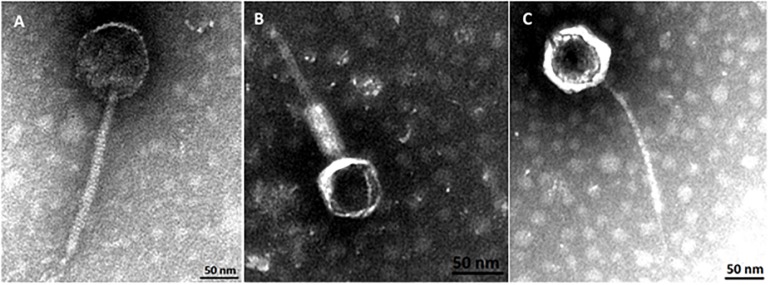
Virion morphology of phages, **(A)** vB_EcoS-2004IV (*Siphoviridae*), **(B)** vB_EcoM-12474V (*Myoviridae*), and **(C)** vB_EcoS-26175V (*Siphoviridae*).

Phylogenetic analysis corroborates that three groups of phages where identified in this study. Transmission electron micrographs from phages vB_EcoM-12474V, vB_EcoS-26175V, and vB_EcoS-2004IV show phages with icosaheadral heads and long tails (Figure 8). Phage vB_EcoM-12474V belongs to the *Myoviridae* family and has a head of about 62 nm and a 155 nm contractile tail. Phages vB_EcoS-2004IV and vB_EcoS-26175V are both siphoviruses and have long non-contractile tails of 232 and 186 nm, respectively. Phage heads have an approximate diameter of 100 and 85 nm.

**FIGURE 9:**
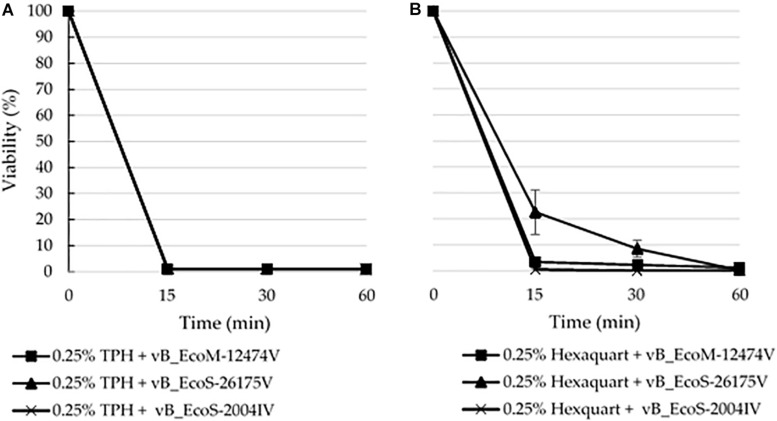
Disinfectant stability of the phages vB_EcoM-12474V (MK907239), vB_EcoS-26175V (MK907271) and vB_EcoS-2004IV (MK907241) in the presence of 0.25% **(A)** TPH Protect and **(B)** Hexaquart^®^ plus.

The lytic capacity of phages from the different groups was investigated using enterobacterial collection strains, clinical isolates and *E. coli* isolated from the samples (Table 2). *Peduovirus* vB_EcoM-12474V, *Tequintavirus* vB_EcoS-26175V, and *Tunavirinae* vB_EcoS-2004IV showed different lysis patterns. None of the isolated phages lysed the bacteria isolated from the blood, urine and tracheal aspirates samples or other clinical isolates previously collected in the hospital setting. Whilst vB_EcoS-26175V could infect the six laboratory strains tested, vB_EcoS-2004IV could only infect DSM 12242, JM109, W3110, and MC1061. The narrower host range was obtained for vB_EcoM-12474V, which only infected two lab strains. This might indicate that the phages are either strain-specific or that the bacteria already acquired resistance to infection (in the case of the bacteria from the native sample). Bioinformatic searches in *Enterobacteriaceae* genomes deposited in the NCBI database revealed a 98–99% sequence identity of the *Peduovirus* isolated during this study with six *E. coli* isolates: STEC O145:H28 strain 95-3192 (CP027362), non-O157 STEC FHI82 (LM996779), STEC O145:H28 strain RM12581 (CP007136), STEC O145:H28 strain RM13514 (CP006027), STEC O145:H28 strain 2015C-3125 (CP027763), and STEC O145 strain RM9872 (CP028379). *Tequintavirus* vB_EcoS-26175V and *Tunavirinae* phage vB_EcoS-2004IV were not detected in previously sequenced bacteria deposited in the database. The phage stability in the presence of the disinfectants TPH Protect and Hexaquart^®^ plus was investigated (Figure 9). A concentration as low as 0.25% TPH Protect was sufficient to inactivate all phage particles of the three phage groups described, after 15 min. After incubation with Hexaquart^®^ plus for 60 min *Peduovirus* vB_EcoM-12474V and *Tequintavirus* vB_EcoS-26175V virions could still be isolated. *Tunavirinae* vB_EcoS-2004IV could no longer be detected after 30 min.

## Discussion

In this study, we were able to successfully isolate natural-occurring phages from different human sources and further characterize the phage community found not only at the morphological level, but also at the nucleotide level. Coliphages that were able to infect the host strain *E. coli* DSM 12242 were present in 14.4% of the samples analyzed in this study. This prevalence is below what was reported in a previous study that surveyed the presence of phages, mainly siphoviruses in human blood, ascitic fluid, urine, cerebrospinal fluid, and serum (>45% of abdominal fluid and urine) (Brown-Jaque et al., 2016). The successful isolation of virions from clinical samples mostly relies on the use of large volumes of human body fluid samples. Despite the high level of sensitivity of *E. coli* DSM 12242 to detect bacteriophages, as previously shown (International Organization for Standardization, 2000; Shousha et al., 2015; Hilbert et al., 2017), we assume that some of the coliphages present in the samples analyzed might have been missed due to a narrow host range (e.g., strain specific) and to the low phage densities despite the use of liquid cultures as an enrichment method. Additionally, some of the phages that are able to infect the strain used may have reduced plating efficiency during the first passage (Weinbauer, 2004).

Human body sites are not as sterile as previously thought and data from phage diversity studies showed that this diversity tends to be very different between ecological niches. The amount of uncharacterized, unknown, and abundant phage populations described in the late years in metagenomics studies highlighted the emergent need to characterize bacteriophages and disclose their role in human health and disease (Hyman and Abedon, 2012). Previous work conducted in our lab highlighted the important role of phages in the transfer of antimicrobial resistance genes through transduction mechanisms in other environments (Shousha et al., 2015; Hilbert et al., 2017). We chose a culture-based method in order to be able to study transduction instead of a metagenomics approach that would only provide information about genes but not the mechanism and ability of transfer. Moreover, the majority of the metagenomics studies cannot classify phages due to lack of homolog sequences in the databases and the so-called “viral dark matter” remains in obscure (Breitbart et al., 2003; Willner et al., 2009; Pride et al., 2012; Dinakaran et al., 2014; Dutilh et al., 2014; Ma et al., 2018).

Studies on the phage distribution in the human body are quite scarce, especially in a clinical context (Brown-Jaque et al., 2016; Navarro and Muniesa, 2017). The vast majority of studies available are mainly focused on the human gut (Górski and Weber-Dabrowska, 2005; Górski et al., 2006; Manrique et al., 2017; Ma et al., 2018) and feces (Havelaar et al., 1986; Cornax et al., 1994; Furuse et al., 2019), with other studies describing phage colonization of the mouth (Ly et al., 2014; Naidu et al., 2014) and respiratory tract (Willner et al., 2009; Willner and Furlan, 2010).

Phages were previously expected to be found everywhere their bacterial host is present, but recent studies suggest that bacterial abundance does not necessarily predict the relative abundance of their phages (Ly et al., 2014; Naidu et al., 2014; Brown-Jaque et al., 2016). The observation of phages at body sites where the bacterial host was not detected gives strength to the possibility of phage translocation through the human body (Górski et al., 2006; Brown-Jaque et al., 2016; Tetz and Tetz, 2018). As a matter of fact, in this study coliphages and *E. coli* were only found concomitantly in five samples. In 11 other samples, phages were found despite the non-detection of the bacterial host.

Phage genomes are characterized by a rapid evolutionary rate, horizontal gene transfer events and recombination between different viral genes, which makes the inference of phylogenetic relationships more reliable when considering individual genes or modules instead of whole genomes (Zois et al., 2011). This work relied on whole-genome analysis (VICTOR), gene content (multi-sequence alignment) and single-gene analysis (signature genes with no signs of recombination) to better disclose the phylogenetic relationship between these phages and other phages from the viral databases. We were able to successfully isolate and characterize 43 phages belonging to three different species (Figure 2). There was a surprising low level of diversity observed, regarding the taxonomical distribution of phages isolated in our study. Phages belonging to the *Peduovirus*, *Tequintavirus*, and *Tunavirinae* subfamily were present in 16 samples. Interestingly, *Peduovirus* and *Tequintavirus* were isolated from a single sample, whilst the *Tunavirinae* were found across several samples. All of the phages were very similar, reflecting a low level of intra-sample diversity. The same can be said about the inter-sample diversity observed for the *Tunavirinae*, which was in fact unexpected. This low level of diversity might be mainly due to the ability of specific phages to survive and multiply in the biological materials tested given the challenges that they present to the phage stability, such as e.g., high and/or low pH conditions (Weinbauer, 2004). Moreover, enteric bacteria and coliphage in pure urine were found to be rapidly inactivated in stored urine, being highly dependent on its pH (Chandran and Pradhan, 2009). The most similar phage genomes in the NCBI Viruses database was compared with the representative of each phage genome sequenced in this study (Supplementary Figures S2, S4, S6). Annotation of the features of the phage nucleotide sequences showed that these are conserved throughout the genomes, with only some genomic rearrangements occurring, according to the different modules that characterize each species analyzed. The vast majority of the phage sequences were structural proteins, with a part of hypothetical proteins, which were further investigated using blastp searches in the viral database for homology in other phage species. This strategy allowed the association of the majority of the hypothetical proteins as phage-related. Moreover, no features associated with antibiotic resistance or virulence were identified in any of the genomes.

When testing these phages for the host range, they could only infect *E. coli* laboratory strains DSM 12242, ATCC 11303, JM109, W3110, MC1061, and DH5α, having no activity against other *Enterobacteriaceae* or other *E. coli* clinical isolates tested (Table 2). These observations highlight the importance of using an adequate host strain for phage isolation, as the use of different *E. coli* strains to analyze the same set of samples has previously shown to yield different *Siphoviridae* coliphages (Chibani-Chennoufi et al., 2004).

The genus *Peduovirus*, together with *Hpunavirus*, compose the *Peduovirinae* subfamily, family *Myoviridae* according to the ICTV. *Peduovirus* vB_EcoM-12474V shared a 97.30% ANI with phage fiAA91-ss, a recognized member of the *Peduovirus* genus previously isolated from an *E. coli* O157:H7 strain from urban raw wastewater (Allué-Guardia et al., 2013). The sequence characteristics are also in accordance with the phage genomes already described for members of this genus, which range between 29.5 and 40.6 Kb and have 40–60 CDS. The classification of these phages as members of the *Peduovirus* genus is also supported by the presence of signature genes as the regulatory region containing the integrase gene *int*, the lytic-lysogenic transcriptional switch genes *cox* (lytic repressor gene) and *C* (immunity repressor gene), and the P2 type of late genes – capsid scaffold gene *O*, major capsid precursor gene *N*, small terminase subunit gene *M* and capsid completion gene *L* (Supplementary Data S2). These genes control the central mechanism of integration/excision in the host genome and the life cycle after infection. The major difference between the vB_EcoM-12474 phages and fiAA91-ss relies on the absence of the cytolethal toxin genes (Allué-Guardia et al., 2013). Altogether, this suggests that the four phages are indeed part of the *Peduovirus* genus, within a previously existing species. Previous works attempted to detect the presence of natural phages in human or animal blood without success (Gaidelyte et al., 2007; Brown-Jaque et al., 2016; Navarro and Muniesa, 2017). They are thought to be inherently absent in the blood of healthy individuals and are not expected to occur naturally. In fact, to the best of our knowledge, the only report describing virulent phage isolation from native blood was in septicemic patients (Gaidelyte et al., 2007). The associated bacterial host DNA is usually co-detected, which may suggest the translocation of bacteria harboring phage particles or lysogenized prophages into the bloodstream (Huh et al., 2019). Phage titers were also previously shown to increase with the severity of the symptoms. In patients with leukemic diseases, an increase in coliphage titer and virulent phages (including T-phages) was previously reported (Furuse et al., 2019). In the case of the *Peduoviruses* isolated in this study, the temperate lifestyle and the co-detection of bacteria suggests a case of lysogenized prophages released by the *E. coli* found in the bloodstream. Moreover, these phages can be found as prophage sequences of Shiga toxin-producing *E. coli* (STEC) strains when searching the NCBI bacterial database. Typically, *Peduovirus* are temperate phage-infecting members of the γ-proteobacteria and tend to be similar when infecting the same host (Zois et al., 2011). The absence of bacteriophages in the blood of healthy individuals was also observed when compared with the virome of patients with HIV/AIDS (Li et al., 2012). Other two blood samples contained siphoviruses, with an apparent lytic lifestyle, which might have reached the bloodstream through translocation from other body sites. No evidence of lysogeny was found in the phage genomes, suggesting that these are lytic phages.

Data on the presence of bacteriophages in human urine is scarce (Caldwell, 1928; Thannesberger et al., 2017; Santiago-Rodriguez, 2018). It was for long assumed as sterile until the urethra level and the presence of microorganisms was normally associated with disease. This idea changed with the use of NGS that strongly support the presence of bacterial communities also in healthy individuals. The urine viral community was previously shown to be quite robust, comprising of about 10^7^ virus-like particles (VLPs), both in healthy subjects and patients harboring an urinary tract infection (Santiago-Rodriguez et al., 2015). These urinary viral communities are mainly composed by phages and Human Papillomaviruses (HPV). Phages in human urine are thought to be mostly lysogenic, infecting bacteria from a wide range of phyla, such as Proteobacteria, Bacteroidetes, Firmicutes and Verrucomicrobia (Santiago-Rodriguez et al., 2015; Santiago-Rodriguez, 2018). This same idea of sterility was assumed for the respiratory tract. The respiratory tract of healthy people is believed to be poorly colonized by microorganisms, thus suggesting that no significant phage population is established. However, samples from the sputum of cystic fibrosis patients revealed a core of cystic fibrosis-associated phage, encoding a plethora of virulence factors as adhesins, biofilm-formation and quorum-sensing genes. In contrast, phages collected from the sputum of healthy individuals were highly diverse and most likely resembled environmental communities (Willner et al., 2009; Willner and Furlan, 2010). All the phages detected in urine and sputum during this study show a lack of integrative elements and seem to have a lytic lifestyle.

Dominant phages that persist at an increased number in individuals through an extended period of time showed no significant genome divergence or mutation, suggesting indeed a high genome stability of phage genomes, maybe due to the absence of substantial selective pressure acting on either bacteria or phages (Reyes et al., 2010). Possibly, this might explain why almost no inter-sample variability was found, and why the *Tunavirinae* group described here was widespread in almost all patients. The subfamily *Tunavirinae* belongs to the *Siphoviridae* family and has currently 8 genera classified: *Eclunavirus, Hanrivervirus*, *Rogunavirus*, *Rtpvirus*, *Sertoctavirus*, *Tlsvirus*, *Tunavirus*, and *Webervirus* [plus a non-classified species *Cronobacter* virus Esp2949-1 (NC_019509)]. Members of this subfamily are between 45 and 52 kb and have between 45 and 94 CDS. The highly abundant *Tunavirinae* isolated across our samples are confidently part of this subfamily, and highest homology was found within the *Rtpvirus*. However, analysis of the large subunit terminase suggests that they are closer to the *Rogunavirus.* The closest related phages were all phages isolated in other *Enterobacteriaceae* such as *Yersinia* sp. and *Salmonella* sp. However, the *Tunavirinae* phage vB_EcoS-2004IV appears to be very similar to a campylobacter phage, which unfortunately is still deemed as unverified, which would suggest an unusual level of promiscuity between phages that infect different host families. The closest homolog shares an 87.69% ANI, which is not enough to place the phages within a pre-existent genus, but enough to classify it as a member of the *Tunavirinae* family. Currently the ANI for the demarcation of a new species is at 95% according to the ICTV criteria. Nonetheless, recent studies suggest that a 97% would provide better accuracy for *Tequintavirus*, *Rb69virus* and *Seuratvirus* (Sazinas et al., 2018; Michniewski et al., 2019). In this case, the ANI criteria set to 97% would mean that also the *Tequintavirus* phage isolated in our study would also be a new species within this genus. Phage vB_EcoS-26175V, despite containing a vast abundance of structural proteins, also contained proteins associated with RNA metabolism (JOHFDMOO_00089) and protein degradation (JOHFDMOO_00009).

By surviving disinfection, these phages could be widespread around the clinical setting and in close proximity with the environment of the patients. The survival of phages in this environment might have deleterious consequences on the outcome of infectious diseases, by mediating the acquisition and dissemination of antimicrobial resistance, as observed in other settings (Colomer-Lluch et al., 2011, 2014; Fard et al., 2011; Shousha et al., 2015; Hilbert et al., 2017). The phage stability in the presence of the disinfectants TPH Protect and Hexaquart^®^ plus was investigated, given their common use as hospital disinfectants. In particular, TPH Protect is the disinfectant used in the hospital where the samples were collected, and is used in a final concentration of 0.5% (v/v). None of the phages survived contact with half that concentration. However, upon exposure to 0.25% (v/v) Hexaquart^®^ plus, *Peduovirus* vB_EcoM-12474V and *Tequintavirus* vB_EcoS-26175V could still be detected after 60 min (Figure 9).

The phages isolated during the course of this study and genome comparison analysis provided new insights regarding the phages that inhabit and colonize human body surfaces and revealed the presence of at least one new taxonomic group. *Peduovirus* vB_EcoM-12474V is a pre-existent species, *Tequintavirus* vB_EcoS-26175V might be a new species (depending on the ANI cut-off considered) and *Tunavirinae* vB_EcoS-2004IV represents a novel species. TEM also provided morphological evidence that supports the inclusion of the phages of this study in the above mentioned taxonomic groups (Figure 9). Instead of characterizing the whole population through metagenomics, this approach allowed the isolation and purification of bacteriophages. This work is a very important contribution and a meaningful attempt in the disclosure of the key players behind the viral dark matter observed, their interaction with bacteria found on site and their possible involvement in the health status of the individuals.

## Data Availability Statement

The datasets generated for this study were deposited under the accession numbers PRJEB32459 and PRJNA541793.

## Author Contributions

FH acquired the funding and administered the project. FH and CP conceived and designed the experiments. I-JP, CA, and CP provided the data and collected the samples. CP, MH, and DS performed the experiments. FH, CP, MH, ND, I-JP, CA, JC, and DS contributed with reagents, materials, and analysis tools. ND and MH performed transmission electronic microscopy analysis. CP prepared the original draft and wrote the manuscript. FH, CA, I-JP, JC, and CP reviewed and edited the manuscript.

## Conflict of Interest

The authors declare that the research was conducted in the absence of any commercial or financial relationships that could be construed as a potential conflict of interest.

## Footnotes

1 https://ggdc.dsmz.de/victor.php

2 https://github.com/tseemann/abricate

3 https://www.ezbiocloud.net/tools/ani

4 https://github.com/marbl/Mash

5 http://tree.bio.ed.ac.uk/software/figtree/
