# Supplementary material for: Natural Occurrence of Escherichia coli-Infecting Bacteriophages in Clinical Samples
Source: Front Microbiol. 2019 Oct 31;10:2484. doi: 10.3389/fmicb.2019.02484 (PMC6834657; doi:10.3389/fmicb.2019.02484)
Supplement: Supplementary file 6 [file Table_6.DOCX]

Supplementary Material

# Supplementary Data

Supplementary Data 1. Phage accession numbers PRJNA541793.

Supplementary Data 2. Peduovirus.

Supplementary Data 3. Tequintavirus.

Supplementary Data 4. Tunavirinae.

Supplementary Data 5. Whole-genome comparisons.

# Supplementary Figures and Tables

## Supplementary Figures


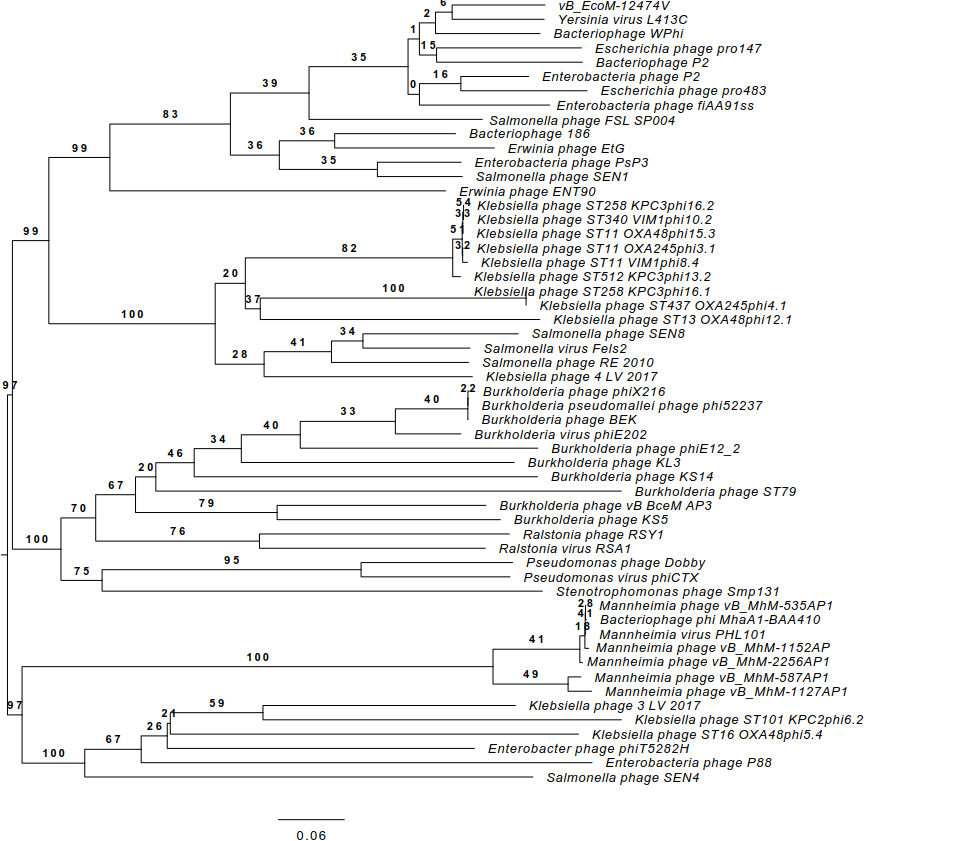


**Supplementary Figure 1.** Phylogenetic tree of the P2 bacteriophages using VICTOR. The scale represents homology % and yields an average support of 49%. *Peduovirus* vB_EcoM-12474V and other 54 complete genomes belonging to this genus extracted from Genbank (April 2019). The numbers above branches are GBDP pseudo-bootstrap support values from 100 replications. Closest homologues are highlighted in green.


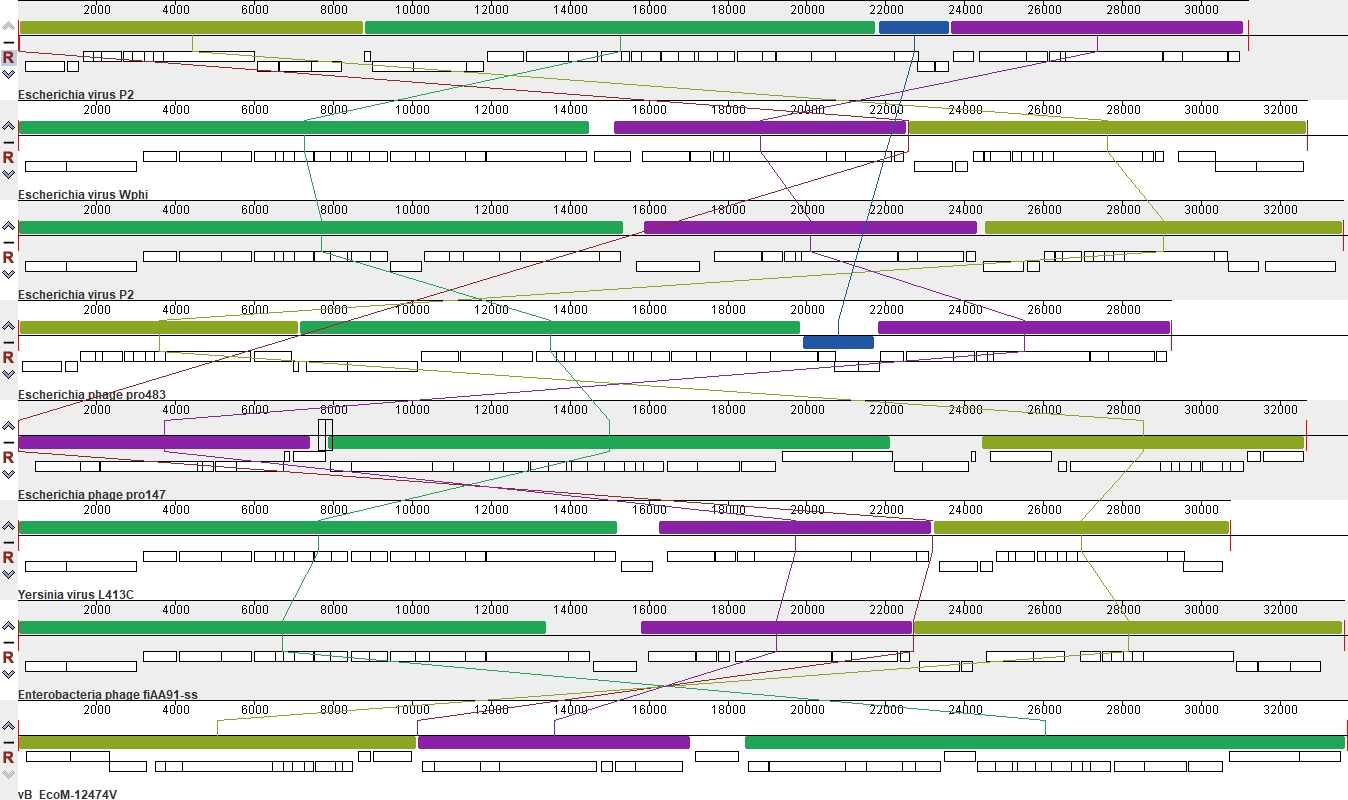


**Supplementary Figure 2.** Genome alignment comparison of phages P2 (NC_001895 and KC618326), Wphi (NC_005056), pro483 (NC_028943), pro147 (NC_028896), L413C (NC_004745), fiAA91ss (NC_022750) and newly-isolated phage vB_EcoM-12474V.


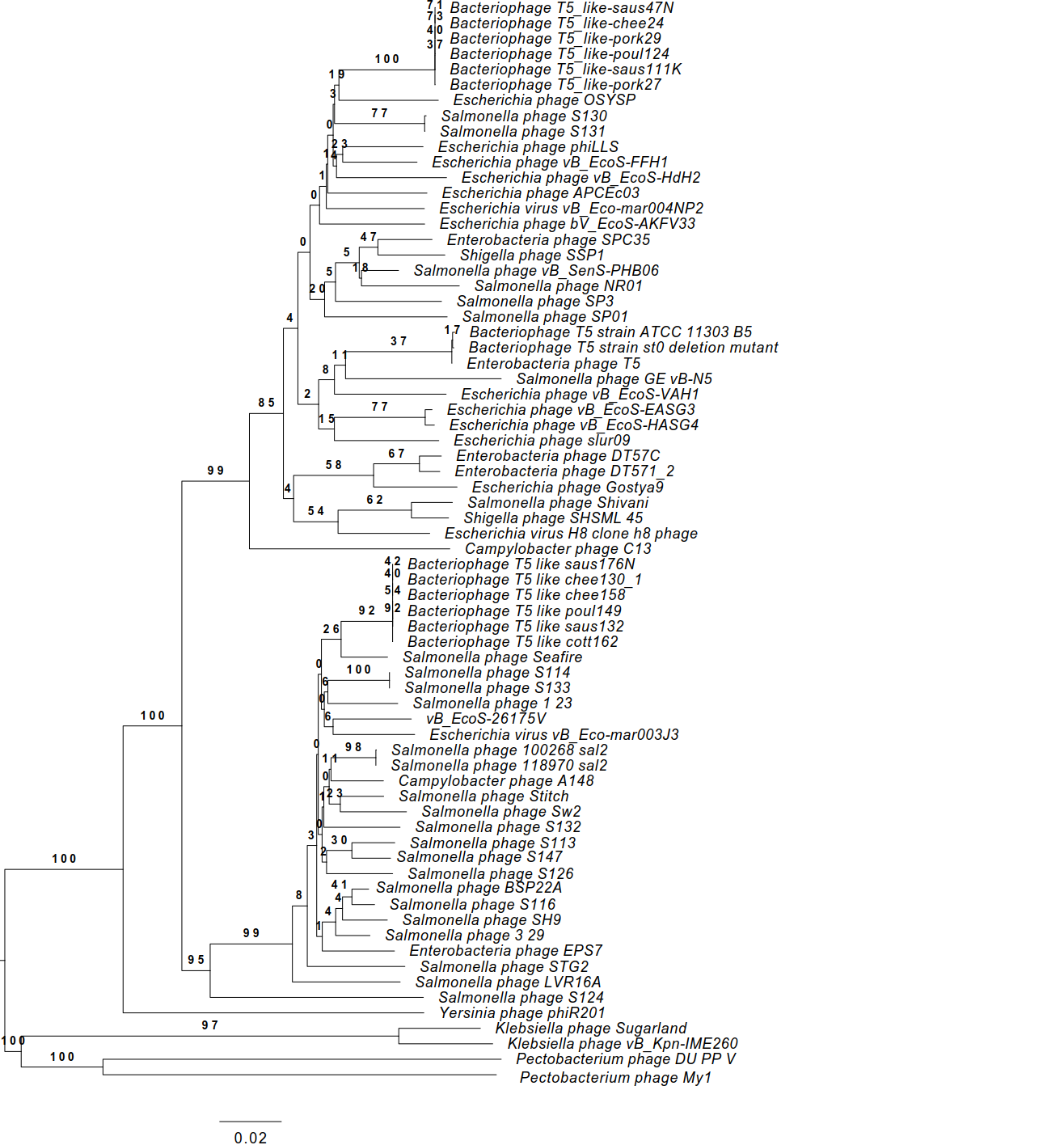


**Supplementary Figure 3.** Phylogenetic tree of the *Tequintavirus* bacteriophages using VICTOR. The scale represents homology % and yields an average support of 36%. *Tequintavirus* vB_EcoS-26175V in blue and other 69 complete genomes belonging to this genus extracted from Genbank (April 2019). The numbers above branches are GBDP pseudo-bootstrap support values from 100 replications. Closest homologues are highlighted in blue.


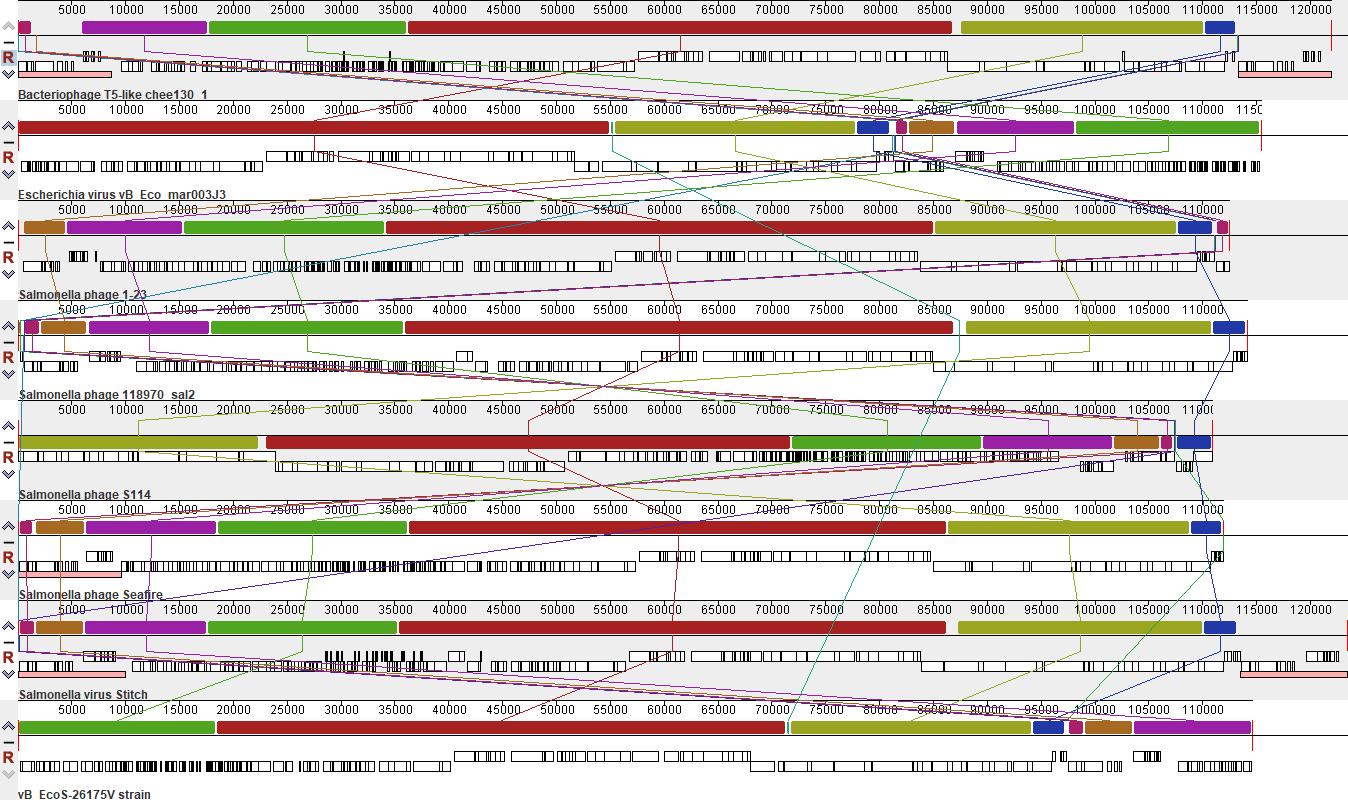


**Supplementary Figure 4.** Genome alignment comparison of phages T5-like chee130_1 (MF431736), vB_Eco_mar003J3 (LR027389), 1-23 (MK370036), 118970_sal2 (NC_0319333), S114 (MH370367), Seafire (MK050846), Stitch (NC_027297) and newly-isolated phage vB_EcoS-26175V.

*Webervirus*

*Hanrivervirus*

*Rogunavirus*

*Rtpvirus*

*Eclunavirus*

*Sertoctavirus*

*Tunavirus*


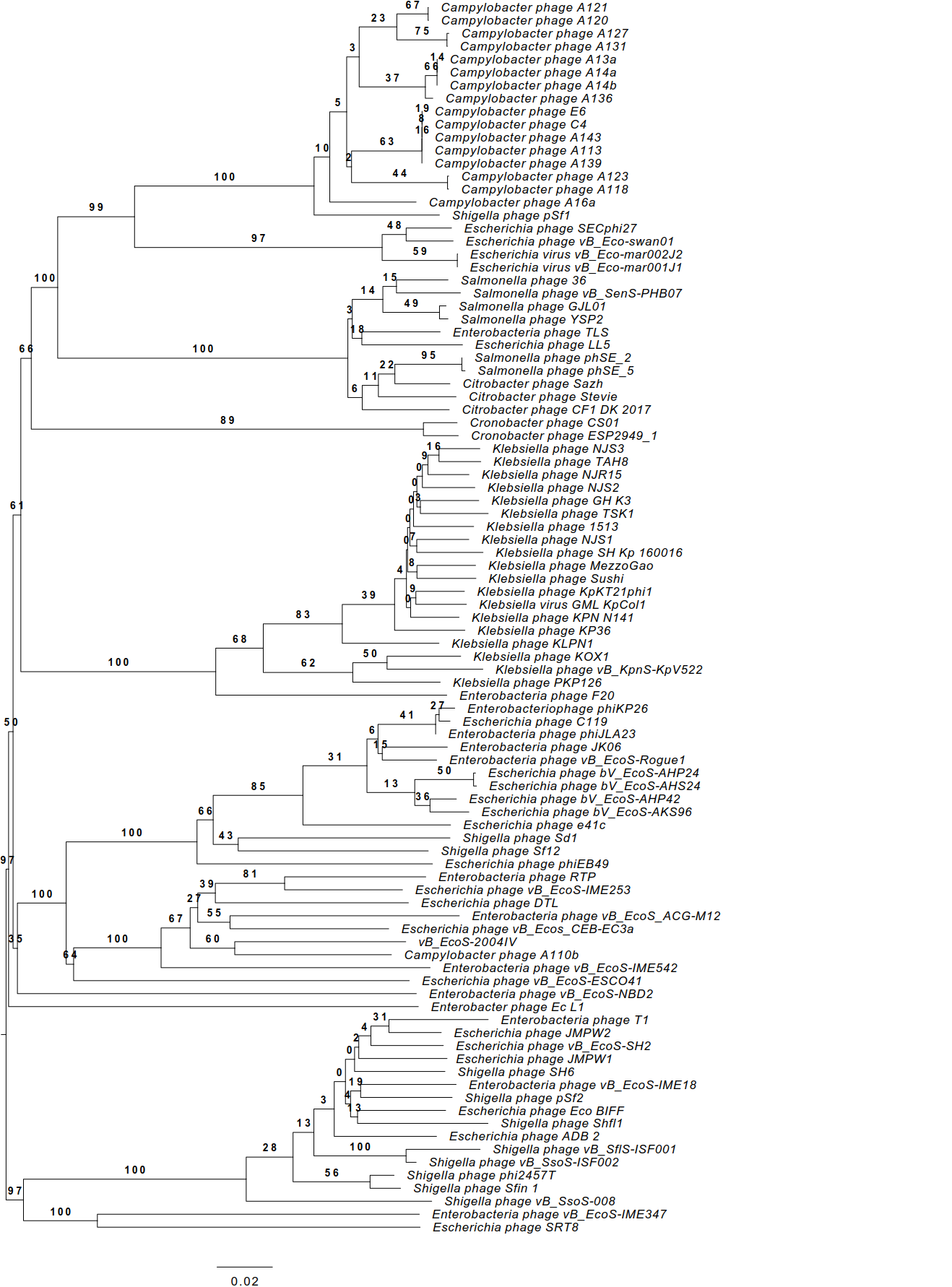


*Tlsvirus*

**Supplementary Figure 5.** Phylogenetic tree of the *Tunavirinae* bacteriophages using VICTOR. The scale represents homology % and yields an average support of 40%. *Tunavirinae* vB_EcoS-2004IV in blue and other 94 complete genomes belonging to this subfamily extracted from Genbank (April 2019*).* The numbers above branches are GBDP pseudo-bootstrap support values from 100 replications. The genera are highlighted in colors.


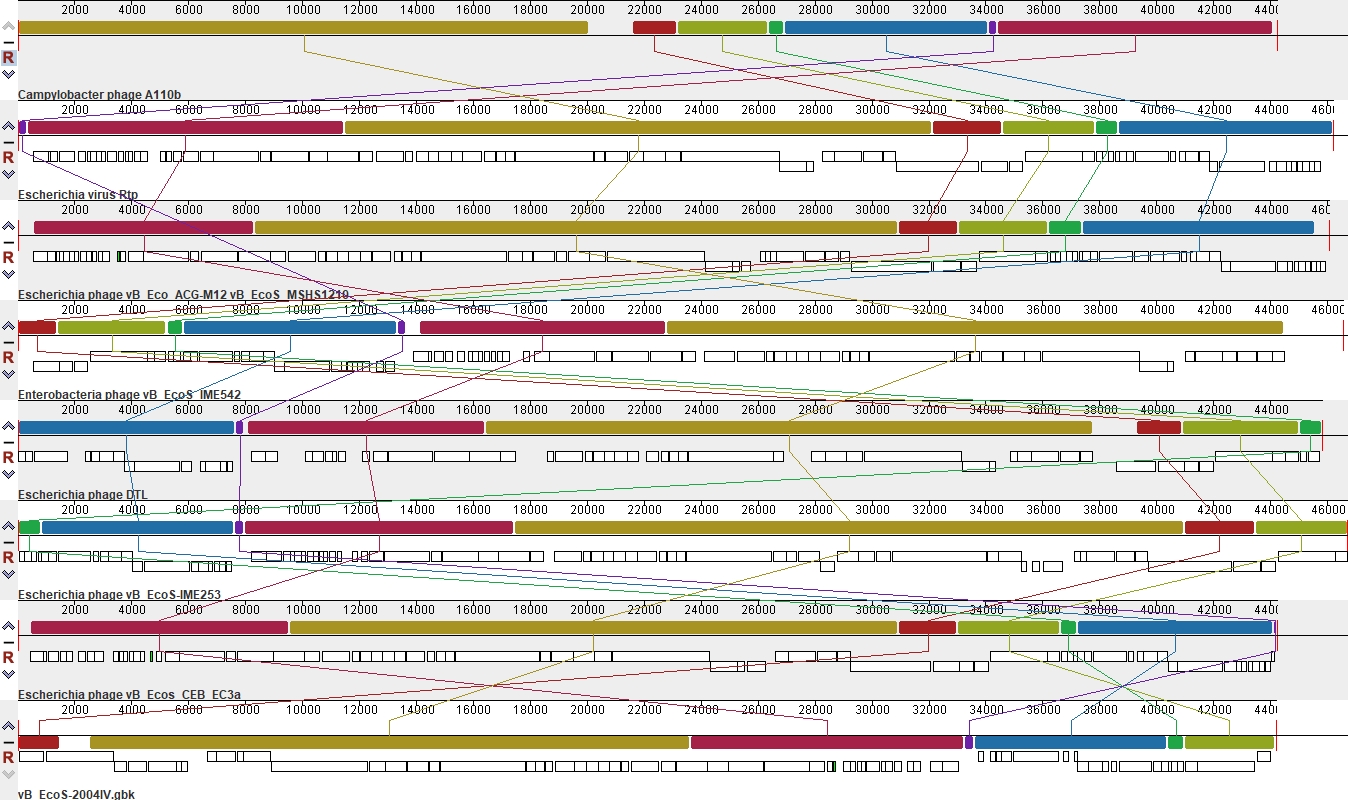


**Supplementary Figure 6.** Genome alignment comparison of phages A110b (MG065688), RTP (NC_007603), vB_Eco_ACG-M12 (NC_019404), vB_EcoS_IME542 (MK372342), DTL (MG050172), vB_Ecos_CEB_EC3a (KY398841), vB_EcoS-IME253 (KX130960) and newly-isolated phage vB_EcoS-2004IV.
